# Prevalence of Various Vaccine Candidate Proteins in Clinical Isolates of *Streptococcus pneumoniae*: Characterization of the Novel Pht Fusion Proteins PhtA/B and PhtA/D

**DOI:** 10.3390/pathogens8040162

**Published:** 2019-09-24

**Authors:** Mitsuyo Kawaguchiya, Noriko Urushibara, Meiji Soe Aung, Masaaki Shinagawa, Satoshi Takahashi, Nobumichi Kobayashi

**Affiliations:** 1 Department of Hygiene, Sapporo Medical University School of Medicine, Hokkaido, Sapporo 060-8556, Japan; noriko-u@sapmed.ac.jp (N.U.); meijisoeaung@sapmed.ac.jp (M.S.A.); nkobayas@sapmed.ac.jp (N.K.); 2 Department of Infection Control and Laboratory Medicine, Sapporo Medical University School of Medicine, Hokkaido, Sapporo 060-8556, Japan; shinagawa@sapmed.ac.jp (M.S.); stakahas@sapmed.ac.jp (S.T.)

**Keywords:** *Streptococcus pneumoniae*, serotype, vaccine candidate proteins, pneumococcal histidine triad protein (Pht), Pht fusion proteins

## Abstract

Pneumococcal proteins unrelated to serotypes are considered to be candidates of antigens in next-generation vaccines. In the present study, the prevalence of vaccine candidate protein genes, along with serotypes and antimicrobial resistance determinants, was investigated in a total of 57 isolates obtained from a tertiary care hospital in Japan. All of the pediatric isolates and 76.6% of the adult isolates did not belong to PCV13 (a 13-valent pneumococcal conjugate vaccine) serotypes, and 70.2% of all isolates showed multidrug resistance. All of the isolates had *ply*, *pavA*, *nanA*, and *nanB*, and high prevalence was noted for the *pspA* and *pspC* genes (96.5% and 78.9%, respectively). Detection rates for the pneumococcal histidine triad protein (Pht) genes *phtA*, *phtB*, *phtD*, and *phtE* were 49.1%, 26.3%, 61.4%, and 100%, respectively. Two fusion-type genes, *phtA/B* and *phtA/D*, were identified, with a prevalence of 36.9% and 14.0%, respectively. These fusion types showed 78.1–90.0% nucleotide sequence identity with *phtA*, *phtB*, and *phtD*. The most prevalent *pht* profile was *phtA* + *phtD* + *phtE* (26.3%), followed by *phtA/B* + *phtE* (19.3%) and *phtA/B* + *phtD* + *phtE* (17.5%), while *pht* profiles including *phtD* and/or *phtA*/*phtD* were found in 71.9% of isolates. The present study revealed the presence of two fusion types of Pht and their unexpectedly high prevalence. These fusion types, as well as PhtA and PhtB, contained sequences similar to the B cell epitopes that have been previously reported for PhtD.

## 1. Introduction

*Streptococcus pneumoniae* (pneumococcus) occasionally causes both invasive and noninvasive pneumococcal disease (IPD and non-IPD, respectively), such as sepsis, meningitis, and community-acquired pneumonia [1], while this bacterium colonizes and persists on the human nasopharynx [2]. Pneumococcal diseases are considered preventable by vaccine. The capsular polysaccharide (CPS) is a principal virulence factor of *S. pneumoniae* and an essential component of commercially available vaccines against pneumococcal infections [3]. Currently, two classes of pneumococcal vaccines, a 23-valent pneumococcal polysaccharide vaccine (PPSV23) and 7-, 10-, and 13-valent pneumococcal conjugate vaccines (PCVs), both of which contain CPS as an immunogen, have been introduced in many countries. The unconjugated PPSV23 is widely offered to only adults because of poorer immunogenicity in children under 2 years of age [4,5], whereas the recently approved PCVs have been used in routine childhood vaccination programs across the world since 2000 [6].

Routine immunization with PCVs in children has greatly reduced the infections caused by pneumococci through the serotypes included in the vaccine (vaccine serotypes). However, following the introduction of PCV7 and PCV13 in children, the isolation rate of pneumococci with nonvaccine types increased globally as a result of the vaccine selection pressure, and recent studies in different countries have reported the occurrence of immediate chronological changes in serotypes [7,8,9]. In Japan, PCV7 was introduced in 2010 and was replaced with PCV13 in 2013. Our previous surveillance studies demonstrated that the rate of non-PCV13 serotypes in pediatric isolates increased from 39.7% in 2011 to 87.9% in 2016 [10,11]. The high prevalence rate of non-PCV13 serotypes was also documented in pediatric carriage isolates in recent studies [12,13]. Moreover, the widespread implementation of PCVs in children has been associated with the emergence and spread of drug-resistant clones with nonvaccine serotypes [14,15,16], posing a major public health concern. The currently available pneumococcal vaccines show protection against infections solely due to serotypes included in the vaccine, i.e., vaccine serotype-specific immunity, which is considered to be a limitation of the vaccines. For these reasons, the development of new pneumococcal vaccines in which the protective effect does not depend on serotypes has been anticipated. 

Multiple pneumococcal proteins have been comparatively well investigated as promising targets for future non-serotype-specific protein-based pneumococcal vaccines, such as pneumococcal surface protein A (PspA) [17,18], pneumococcal surface protein C (PspC) [19,20], pneumococcal choline-binding protein (PcpA) [21], three neuraminidases (NanA, NanB, and NanC) [22,23], pneumolysin (Ply) [24], and four pneumococcal histidine triad proteins (PhtA, PhtB, PhtD, and PhtE) [25,26,27,28]. At present, an investigational pneumococcal vaccine is undergoing Phase I/II clinical trials in infants [29,30], children [31], and adults [32]. In these studies, two pneumococcal proteins, Ply toxoid (dPly) and PhtD, are included in the vaccines. A protein-based pneumococcal vaccine containing PhtD–dPly was shown to induce protection against pneumococcal pneumonia in a rhesus macaque study [33]. All four Pht proteins were exposed on the cell surface of pneumococcus and were characterized by histidine triad motifs in their amino acid sequences [34]. Among them, PhtD was described to be the least genetically variable [35] and was shown to induce an immune response in adults and infants [36], with immunodominant B cell epitopes being identified [27]. In addition to the PhtD protein, previous studies have indicated the presence of hybrid types of Pht in pneumococcus between PhtA and PhtB and between PhtA and PhtD [34,35]. However, genetic organization and sequences of the Pht hybrid/fusion types in *S. pneumoniae* have not yet been clearly described, and the prevalence of the hybrid types among clinical isolates remains to be determined.

The purpose of the present study was to investigate the prevalence of vaccine candidate protein genes in clinical isolates of *S. pneumoniae* collected from IPD and non-IPD patients, along with serotypes and antimicrobial resistance. Particularly, we focused on an identification and genetic analysis of the Pht fusion types that have not been well characterized, as well as the known Pht types. Consequently, we reveal the existence of two unique fusion types (PhtA/B and PhtA/D) in various serotypes of *S. pneumoniae*. The prevalence and profiles of authentic Pht and Pht fusion types in the clinical isolates are described in parallel. 

## 2. Results

### 2.1. Serotypes and Sequence Types (STs) of Pneumococcal Isolates

For the 57 isolates analyzed, 21 different serotypes and 33 different STs, including two new STs, were identified. Serotypes and STs of all the isolates are listed in Table 1 with the designation of Pneumococcal Molecular Epidemiology Network (PMEN) international clones. All of the pediatric isolates belonged to non-PCV13 serotypes, i.e., 15B (ST199; Netherlands^15B^-37 PMEN clone), 23A (ST338; Colmbia^23F^-26 PMEN clone or its single-locus variant (SLV)), 24F (ST2572 or its SLV), or 35B (ST558, ST2755). Among isolates from adults, the three most prevalent serotypes were 15A (mostly ST63; Sweden^15A^-25 PMEN clone or its SLV, ST292 SLV), 3 (mostly ST180; Netherlands^3^-31 PMEN clone), and 35B (ST558, ST2755). Serotypes covered by PCV13 and PPSV23 in all adults were 23.4% (*n* = 11/47) and 44.7% (*n* = 21/47), respectively. 

### 2.2. Detection of Drug Resistance Genes and Antimicrobial Susceptibility

The antimicrobial susceptibility of all the isolates with individual serotypes is shown in the Supplementary Materials, Table S1. The highest nonsusceptibility rates were observed for erythromycin (91.2%) and tetracycline (86.0), which corresponded to the high prevalence of resistance genes to macrolides (*erm*(B) and/or *mef*(A/E)) and tetracycline (*tet*M) (94.7% and 93.0%, respectively). Nonsusceptibility to penicillin was detected in 38.9% of isolates, among which two isolates (serotypes 6C and 15A) were resistant to penicillin. All of the isolates showing nonsusceptibility to penicillin had the penicillin-binding protein (PBP) genotype penicillin-resistant *S. pneumoniae* (gPRSP) (alterations in three genes: *pbp1a*, *pbp2x*, and *pbp2b*). The prevalence of the multidrug resistance (MDR) phenotype (defined as resistance to three or more different classes of antibiotics) was 70.2%. Three MDR isolates (5.3%, 3/57) belonging to serotypes 14 (*n* = 1) and 15A (*n* = 2) were resistant to levofloxacin, which is associated with mutations in the quinolone resistance-determining region (QRDR) (positions of mutations are shown in the Supplementary Materials, Table S1, footnote). 

### 2.3. Detection of the Vaccine Candidate Pneumococcal Protein Genes and the Novel Pht Fusion Proteins

The prevalence of various pneumococcal proteins among individual serotypes is summarized in Table 2. All of the isolates carried the *ply* and *pavA* genes. The prevalence of the *pspA* and *pspC* genes was 96.5% and 78.9%, respectively. Among the three *nan* genes, *nanA* and *nanB* were detected in all of the isolates, while *nanC* was in 52.6% of the isolates. Detection rates for *phtA*, *phtB*, *phtD*, and *phtE* were 49.1%, 26.3%, 61.4%, and 100%, respectively. In addition to the four *pht* genes, two fusion-type genes, *phtA/B* and *phtA/D*, were identified, with the prevalence at 36.9% and 14.0%, respectively. Two serotype 15B isolates had both *phtD* and *phtA/D*. 

### 2.4. Prevalence of the Pht Pattern and Sequence Analysis of the Pht Fusion Types

**Figure 1:**
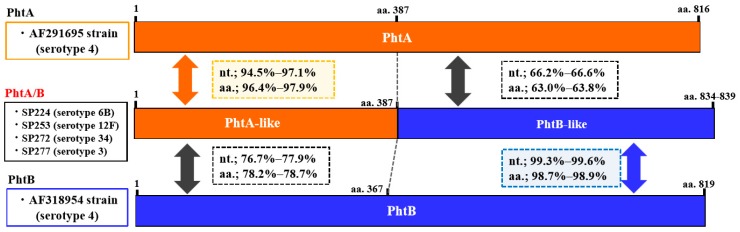
Genetic structure of the *phtA/B* (fusion type of *phtA* and *phtB*) gene identified in the present study and *phtA* and *phtB* in reference strains (GenBank Accession Nos. AF291695 and AF318954, respectively). The serotype of each isolate is indicated in parentheses. The *phtA/phtA*-like and *phtB/phtB*-like sequences are shown in orange and blue, respectively. Nucleotide (nt.) and amino acid (aa.) sequence identities of *phtA*-like and *phtB*-like regions of *phtA/B* gene with those of *phtA* and *phtB* genes are shown in squares with dotted lines.

**Figure 2:**
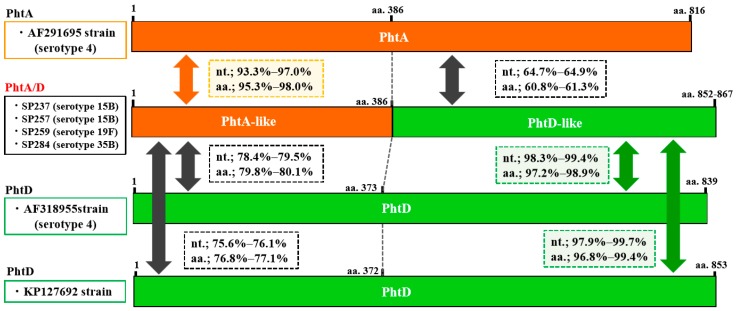
Genetic structure of the *phtA/D* (fusion type of *phtA* and *phtD*) gene identified in the present study and *phtA* and *phtD* in reference strains (GenBank Accession Nos. AF291695, AF318954, and KP127692, respectively). The serotype of each isolate is indicated in parentheses. The *phtA/phtA*-like and *phtD/phtD*-like sequences are shown in orange and green, respectively. Nucleotide (nt.) and amino acid (aa.) sequence identities of *phtA*-like and *phtD*-like regions of *phtA/D* gene with those of *phtA* and *phtD* genes are shown in squares with dotted lines.

*Pht* profiles are summarized in Table 3. The most prevalent *pht* pattern was *phtA* + *phtD* + *phtE* (26.3%), followed by *phtA/B* + *phtE* (19.3%), *phtA/B* + *phtD* + *phtE* (17.5%), and *phtA* + *phtB* + *phtD* + *phtE* (14.0%). *Pht* gene profiles, including *pht* fusion type, were detected in 50.9% of all isolates. Structural organizations of the fusion types PhtA/B and PhtA/D (compared to PhtA, PhtB, or PhtD) are schematically represented in Figure 1 and Figure 2, respectively. The sequence alignment of these genes is shown in the Supplementary Materials, Figures S1 and S2. These fusion-type Pht proteins were constituted by the N-terminal half of the *phtA*-like region and the *phtB*- or *phtD*-like region in the C-terminal side. The *phtA*-like region of *phtA/B* from the four isolates (aa.1–387) had 94.5–97.1% sequence identity with *phtA* in a reference strain (AF291695) and 76.7–77.9% identity with *phtB* in a reference strain (AF318954). In contrast, the *phtB*-like region (aa.387–) of *phtA/B* showed 99.3–99.6% identity with the *phtB* sequence. Similarly, *phtA/D* of the four representative isolates showed 93.3–97.0% identity with *phtA* (AF291695) in the *phtA*-like region and 97.9–99.7% identity with *phtD* (AF318955 and KP127692) in the *phtD*-like region. The nucleotide sequence identity within *phtA/B* was 96.6–98.4%, and similarly, 97.6–97.9% identity was found within *phtA/D* (Supplementary Materials, Table S2). In the present study, nucleotide sequences of *phtB* were determined for five isolates (Supplementary Materials, Table S3), which showed 97.6–99.4% identity with the *phtB* gene reference strain AF318954. In addition, *phtA* had 69.2–70.5% identity with *phtB* and *phtD*; 92.6–93.0% identity was found between *phtB* and *phtD*; and *phtA/B* and *phtA/D* had 78.1–90.0% identity with *phtA*, *phtB*, and *phtD*. 

### 2.5. Sequences of B Cell Epitopes in PhtA, PhtB, PhtD, and Pht Fusion Types

Immunodominant B cell epitopes in PhtD have been identified by Lagousi and coworkers [27] and have been mapped into three regions, i.e., amino acids 88–107 (epitope I, pep11), 172–191 (epitope II, pep17), and 200–219 (epitope III, pep19). The alignment of Pht amino acid sequences indicated that the three epitopes were highly conserved in different Pht proteins, including the fusion types (Supplementary Materials, Figures S1–S3). In particular, epitopes I and III showed almost identical sequences, with only a few substitutions with similar amino acids.

## 3. Discussion

In the present study, we investigated the prevalence of 15 genes in promising vaccine candidate proteins and revealed the existence of two Pht fusion types besides the four known Pht proteins in pneumococcus. To our knowledge, this is the first report to show the prevalence of the fusion types PhtA/B and PhtA/D in various serotypes of pneumococcal isolates. 

At present, for protein-based vaccines, pneumococcal proteins such as PhtD [37], dPly [38], PspA [39], and PcpA [40] have been proven to show effective protection against pneumococcal diseases in studies on human subjects. Further, a pneumococcal protein-based vaccine containing the PhtD protein and/or dPly has been investigated in a Phase I/II randomized clinical study [29,30,31,32]. In our present study, while the prevalence of *ply* was high (100%), *phtD* was detected in 61.4% of all isolates. A similar prevalence of *phtD* (61.0%) was reported in pneumococcal isolates from meningitis in France [41]. In contrast, Rioux et al. reported a high prevalence of *phtD* (100%) in the pneumococcal strains analyzed [35]. In the present study, we identified the Pht fusion types in clinical isolates of pneumococcus, which were classified into two distinct types, PhtA/B and PhtA/D. The prevalence of *phtA/B* and *phtA/D* was 36.9% and 14.0%, respectively, and *phtD* and/or *phtA/D* were found in 71.9% of all the isolates. Although the number of isolates analyzed in the present study was not sufficient, the prevalence of *phtD* and/or *phtA/D* was considered to be high (>70%). The prevalence of *phtD* (*phtA/D*) may vary depending on study subjects (e.g., serotypes/genotypes of the clinical isolates, country, infection types) and will be further clarified if a detection method for the fusion type *phtA/D* is established. 

Although only two studies have described the hybrid/fusion types of Pht in pneumococcus to date [34,35], their sequence data have not yet been published. In our present study, sequences and genetic organizations of the hybrid types of *pht* genes were revealed for the first time, and these were found to be distributed to half of the isolates, including 13 different serotypes. Furthermore, in a BLAST search, we found that the sequences of *phtA/B* (strain SP224, serotype 6B) and *phtA/D* (strain SP284, serotype 35B) in the present study were similar to those in the *S. pneumoniae* complete genome of strain G54 (serotype 19F, GenBank Accession No. CP001015) (99.2% identity) [42] and strain Sp99_4038 (serotype 3, GenBank Accession No. FQ312041) (98.4% identity) [43], respectively. These findings suggest that the fusion types of Pht may be commonly distributed to clinical isolates of pneumococci with various serotypes. 

Adamou et al. first reported pneumococcal *pht* genes [44] and described PCR primers to detect *phtA*, *phtB*, *phtD*, and *phtE*, which were used for their detection in a previous study [41]. However, hybrid types are not detected by uniplex PCR with these primers, and it is also possible that the hybrid types may be misclassified as *phtA*, *phtB*, or *phtD* by nonspecific PCR amplification due to sequence diversity in the *pht* genes. In contrast, there have been few studies that have determined full-length *pht* genes that determine Pht types. These may be possible reasons that fusion-type Pht has been rarely reported. 

The most significant finding in the present study is that the fusion types PhtA/B and PhtA/D had almost identical sequences to B cell epitopes that have been reported for PhtD previously [27], despite overall sequence diversity in Pht. This finding suggests that *S. pneumoniae* with the fusion type Pht may also be recognized by antibodies to PhtD. These B cell epitopes of PhtD are also conserved in PhtA and PhtB [27]. In the present study, 26.3% of isolates (*n* = 15) had only fusion-type *pht* genes (*phtA/B* or *phtA/D*), except for *phtE*. These isolates could be judged as negative for *phtA*, *phtB*, and *phtD* by using the previously reported PCR scheme [41] as described above, and therefore these isolates are not regarded as being protected by an immune response to PhtD. However, our present study revealed that all of the *S. pneumoniae* isolates examined (belonging to various serotypes) possessed one or more of *phtA*, *phtB*, *phtD*, *phtA/B*, or *phtA/D*. This finding may suggest the possibility that PhtD is useful as a broadly protective pneumococcal vaccine. 

In the present study, the most common serotypes were 15A, 3, 6C, and 35B. The prevalence of MDR was 70.2%, and high rates of nonsusceptibility to penicillin were notable for non-PCV13 serotypes 6C, 15A, and 35B. In studies in the United Kingdom [15] and Germany [14], an increase of MDR serotype 15A was observed in the PCV vaccination era, and the major MDR serotypes 15A, 6C, and 35B were noted in the USA [45]. Further, in Canada, the high prevalence has been reported for MDR serotypes 15A and 35B, which are related to the Sweden^15A^-25 PMEN clone and the Utah^35B^-24 PMEN clone, respectively [15]. The recent trends from various countries were also observed in our present study in Japan, suggesting concerns about the dissemination of MDR clones with non-PCV13 serotypes. Taken together, the worldwide spread of non-PCV13 serotypes with multidrug resistance may lead to a limit in the effectiveness of antimicrobial therapy and current vaccination; thus, the development of novel effective vaccines that are irrespective of prevailing serotypes is anticipated. 

The limitations of this study were its small sample size and the fact that most isolates were collected from noninvasive infections or colonization in a single hospital. PhtD is one of the promising vaccine candidate proteins and has been one of the most well studied [25,34,36,37]. In this regard, for basic information, further epidemiological studies are necessary on the prevalence of pneumococcal proteins in clinical isolates, especially Pht proteins, in various regions and countries.

## 4. Materials and Methods 

### 4.1. Pneumococcal Isolates

From March 2016 to February 2018, 57 nonduplicate *S. pneumoniae* clinical isolates from consecutive patients with pneumococcal diseases (either invasive (four isolates from blood) or noninvasive (53 isolates from sputum, nasal discharges, or other nonsterile sites) infections) were collected at the Sapporo Medical University Hospital, Hokkaido, on the northern main island of Japan. Among the isolates studied, 10 and 47 isolates were obtained from children (age < 16 years) and adults (age ≥ 16 years), respectively, and the male/female ratio was 1.1 (27/25). *S. pneumoniae* characteristics were identified by an automated bacterial identification and susceptibility testing system (MicroScan® WalkAway 96 plus system MicroScan; SIEMENS Healthcare Diagnostics) and were confirmed by the detection of the *lytA* gene using PCR, as described previously [46]. Isolates were stored in a Microbank (Pro-lab Diagnostics, Richmond Hill, Canada) at −80 °C. Frozen isolates were inoculated onto a blood agar base supplemented with 5% sheep blood (Nippon Becton Dickinson) and were incubated at 37 °C with 5% CO_2_ for 24 h before further analysis.

In the present study, no human participants were involved directly. Hence, human ethics clearance was not required. We analyzed bacterial isolates as study subjects, which had already been isolated from clinical samples through routine bacteriological examination in our university hospital.

### 4.2. Total DNA Extraction and Sequencing

Genomic DNA was extracted from each isolate as described preciously [47] and was used as a template in all PCR reactions. For determination of the nucleotide sequence, the purified PCR products were sequenced using a BigDye Terminator v3.1 Cycle Sequencing Kit (Applied Biosystems, Foster City, CA, USA) on an automated DNA sequencer (ABI PRISM 3130).

### 4.3. Serotyping, Virulence Gene Detection, and Multilocus Sequence Typing (MLST)

All pneumococcal isolates were subjected to serotyping, virulence gene identification, and genotyping through an MLST scheme. The serotyping of pneumococcal isolates was performed by PCR-based deduction protocols [48] with serogroup/serotype-specific primers (described on the CDC website (http://www.cdc.gov/streplab/pcr.html)). After the PCRs, additional subtyping was performed by PCR-based sequencing methods, as described in our previous studies [11,49,50,51,52]. Fourteen virulence-associated genes, *pht* (*A*, *B*, *D*, and *E*), *pspA* (Family 1, 2, or 3), *pspC*, *pspC*.*4*, *nan* (*A*, *B*, and *C*), *pcpA*, *psrp*, *ply*, and *pavA*, were examined by uniplex PCR with the specific primers reported previously [34,41]. MLST was performed as described on the PubMLST website (http://pubmlst.org/spneumoniae) with modified primers (http://www.cdc.gov/streplab/alt-mlst-primers.html). Subsequently, the obtained STs were compared to Pneumococcal Molecular Epidemiology Network (PMEN) international clones (http://www.sph.emory.edu/PMEN). Allelic numbers/locus sequences of untypable STs were submitted to the PubMLST database curator for assignment of new STs.

### 4.4. Antimicrobial Resistance Determinants

The minimum inhibitory concentrations (MICs) of all isolates against 10 antimicrobial agents (penicillin (PEN), erythromycin (ERY), tetracycline (TET), clindamycin (CLI), trimethoprim-sulfamethoxazole (SXT), ceftriaxone (CRO), cefaclor (CEC), imipenem (IPM), levofloxacin (LVX), and vancomycin (VAN)) were measured through the broth microdilution method using a Dry Plate (Eiken, Tokyo, Japan) as described previously [49] and were interpreted as susceptible (S), intermediate (I), or resistant (R) according to Clinical and Laboratory Standards Institute guidelines (CLSI 2015). The CLSI provides breakpoints as follows: PEN (I = 0.12–1 μg/mL, R ≥ 2  μg/mL), ERY (I = 0.5 μg/mL, R ≥ 1 μg/mL), TET (I = 2 μg/mL, R ≥ 4 μg/mL), CLI (I = 0.5 μg/mL, R ≥ 1 μg/mL), SXT (I = 1/19–2/38 μg/mL, R ≥ 4/76 μg/mL), CRO (I = 2 μg/mL, R ≥ 4 μg/mL), CEC (I = 2 μg/mL, R ≥ 4 μg/mL), IPM (I = 0.25–0.5 μg/mL, R ≥ 1 μg/mL), LVX (I = 4 μg/mL, R ≥ 8 μg/mL), and VAN (S ≤ 1 μg/mL). Multidrug resistance (MDR) was defined as resistance to three or more different antimicrobial agent classes (penicillin resistance was defined using the CLSI breakpoint for oral penicillin V, MIC ≥ 2 μg /mL) [53,54].

For all isolates, alterations of the PBP genes (*pbp1a*, *pbp2x*, and *pbp2b*) and the presence of macrolide (*erm*(B), *mef*(A/E))- and tetracycline (*tet*M)- resistant genes were confirmed by a PCR or multiplex PCR assay [46,55,56]. For three isolates that showed resistance to LVX (MIC of ≥ 8 μg/mL), quinolone resistance-determining region (QRDR) mutations of the DNA gyrase (*gyrA* and *gyrB*) and topoisomerase IV (*parC* and *parE*) genes were investigated by direct sequencing with PCR products, as described previously [57]. 

### 4.5. Detection and Sequence Analysis of PhtA/B and PhtA/D Fusion Types

Fusion types of *PhtA/B* and *PhtA/D* genes were detected, and their sequences were analyzed as follows. First, to identify the fusion type-associated *phtA* gene, all of the isolates were subjected to two PCRs to detect 5′- and 3′-half regions of the *phtA* gene. For these PCRs, two primer pairs were designed on the basis of the published *phtA* sequence (AF291695): PhtA-5’F (5′-ACATCGTGAAGGTGGAACTCC-3′) and PhtA-5′R (5′-GTGTTATCGCTATTTTGTCG-3′) for the 5′-end region (product size 273 bp) and PhtA-3′F (5′-GCCAGTAGAGGAAACACCTGC-3′) and PhtA-3′R (5′-TATCCATAATTTGAAGAGTC-3′) for the 3′-end region (product size 186 bp). The PCR program consisted of the following steps: initial denaturation at 94 °C for 2  min, followed by 35 cycles at 94 °C for 15  s, 55 °C for 150  s, and 72 °C for 15  s, followed by a final extension step at 72 °C for 3  min. Among all isolates, 28 isolates (49.1%) were positive for *phtA* in the two PCRs as well as in the initial PCR for *phtA* gene detection. 

Second, for all of the remaining 29 isolates (50.9%) that had only a 5’-end region of *phtA* (negative for 3’-end region of *phtA*), PCR was attempted with a forward primer of *phtA* (*phtA* primer-F) and reverse primers specific to *phtB* (*phtB* primer-R) or *phtD* (*phtD* primer-R) with the PCR program and conditions described previously [34]. Using the obtained RCR products, *pht* gene sequences were determined through the Sanger method. Finally, 5’-end and 3’-end portions of *pht* gene sequences that were not covered by the above PCR were determined by PCR and direct sequencing using the primers PhtA.F-5’outer (5’-AAGTCCAACCTTGAAAAAGTAGTGG-3’, for *phtA*), PhtB.R-3’outer (5’-GAACTAGAACTCACATTCTGC-3’, for *phtB*), and phtD.R-3’outer (5’-TAACAGCTGATCCAGCTGC-3’, for *phtD*). Sequence data (full-length, 5’-end, and 3’-end half regions) of the presumptive fusion type genes were further analyzed for their highly similar sequences in GenBank (https://www.ncbi.nlm.nih.gov/genbank/) by using BLAST (https://blast.ncbi.nlm.nih.gov/Blast.cgi). Multiple alignments of nucleotide and amino acid sequences for the fusion type *pht* and authentic *phtA*, *phtB*, *phtD*, and *phtE* were performed by the Clustal Omega program (https://www.ebi.ac.uk/Tools/msa/clustalo/), which was used for the calculation of sequence identities between them. In addition, we determined sequences of *phtB* for a representative five isolates because only a few *phtB* genes have been deposited into the GenBank database. 

### 4.6. GenBank Accession Numbers 

The nucleotide sequences of Pht fusion type *phtA/B* and *phtA/D* genes and the *phtB* gene were deposited into the GenBank database under accession numbers MN206792 to MN206804, and they are listed in the Supplementary Materials, Table S3.

## 5. Conclusions

In conclusion, our study revealed the prevalence of 14 vaccine candidate protein genes in clinical isolates of *S. pneumoniae* and demonstrated the existence of PhtA/B and PhtA/D fusion types in various pneumococcal serotypes. These fusion types, as well as PhtA and PhtB, contained sequences of B cell epitopes similar to those previously reported for PhtD, which is included in the investigational protein-based pneumococcal vaccine presently. Despite the small number and limited source of isolates, PhtD and PhtA/D were detected in 61.4% and 14.0% of all isolates. However, all of the isolates with various serotypes had one or more of PhtA, PhtB, PhtD, and fusion types PhtA/B and PhtA/D, suggesting that an immune response to PhtD may confer protective immunity to *S. pneumoniae* irrespective of serotype. Further epidemiological studies on higher numbers of isolates from various sources in various regions are required to determine the prevalence and profiles of *pht* genes among pneumococci as basic information for the development of Pht-based vaccines.

## Figures and Tables

**Table 1:** Serotypes and multilocus sequence types of all the isolates analyzed in this study.

Vaccine Type	Serotype	Children	Adults	Total	ST ^c^ (No. of Isolates)	MLST Allelic Profile ^d^	Remarks
		n = 10	n = 47	n = 57 (%)			(PMEN Clone/Related ST) ^e^
PCV13 ^a^ type	3	0	5	5 (8.8)	180 (4)	7-15-2-10-6-1-22	Netherlands^3^-31
					2331 (1)	10-16-150-1-17-1-29	
	6A	0	2	2 (3.5)	3113 (1)	8-8-4-16-77-1-68	
					7836 (1)	15-29-4-21-6-1-14	
	6B	0	2	2 (3.5)	**14,601** (1)	2-29-4-1-6-121-**11**	SLV of ST5232
					1092 (1)	2-13-2-1-3-19-14	
	19F	0	2	2 (3.5)	257 (1)	**22**-16-19-15-6-20-**14**	DLV of ST236/Taiwan^19F^-14
					236 (1)	15-16-19-15-6-20-26	Taiwan^19F^-14
PPSV23 ^b^ Type (Except PCV13 Type)	10A	1	1	2 (3.5)	5236 * (2)	7-**12**-1-1-10-1-**11**	DLV of ST113/Netherlands^18C^-36
	11A/11D	1	1	2 (3.5)	99 (3)	5-8-4-16-6-1-31	
	12F	0	1	1 (1.8)	4846 (1)	12-32-111-1-13-48-6	
	14	0	2	2 (3.5)	2922 (2)	1-5-4-5-5-**20**-8	SLV of ST9/England^14^-9
	15B	2	2	4 (7.0)	199 (4)	8-13-14-4-17-4-14	Netherlands^15B^-37
	22F/22A	0	4	4 (7.0)	433 * (4)	1-1-4-1-18-58-17	
	33F	0	1	1 (1.8)	717 (1)	5-35-29-1-45-39-18	
Non-Vaccine Type	6C	0	5	5 (8.8)	282 (1)	**30**-4-2-4-4-1-1	SLV of ST81/Spain^23F^-1
					5832 (4)	7-9-4-16-1-6-384	
	6E	0	1	1 (1.8)	90 (1)	5-6-1-2-6-3-4	Spain^6B^-2
	15A	0	8	8 (14.0)	63 * (5)	2-5-36-12-17-21-14	Sweden^15A^-25
					13,065 * (1)	2-5-36-12-17-21-**384**	SLV of ST63/Sweden^15A^-25
					13,068 (1)	2-5-36-12-17-**777**-14	SLV of ST63/Sweden^15A^-25
					**14,602** (1)	7-8-8-8-6-28-**664**	SLV of ST292
	15C	0	2	2 (3.5)	199 (2)	8-13-14-4-17-4-14	Netherlands^15B^-37
	23A	2	0	2 (3.5)	338 (1)	7-13-8-6-1-6-8	Colombia^23F^-26
					8340 (1)	7-**367**-8-6-1-**337**-8	DLV of ST338
	24F	2	0	2 (3.5)	2572 (1)	7-75-9-6-25-6-14	
					5496 (1)	7-257-9-6-25-6-14	
	31	0	1	1 (1.8)	11,184 (1)	1-2-461-16-15-155-18	
	34	0	2	2 (3.5)	3116 (3)	10-8-6-1-9-1-279	
	35B	2	3	5 (8.8)	558 (3)	18-12-4-44-14-77-**97**	SLV of ST377/Utah^35B^-24
					2755 (2)	10-12-2-1-152-28-14	
	37	0	2	2 (3.5)	447 (1)	29-33-19-1-36-22-31	
					7970 (1)	29-33-19-1-36-**482**-31	SLV of ST447

^a^ 13-valent pneumococcal conjugate vaccines (PCV13): 1, 3, 4, 5, 6A, 6B, 7F, 9V, 14, 18C, 19F, 19A, and 23F. ^b^ 23-valent pneumococcal polysaccharide vaccine (PPSV23): 1, 2, 3, 4, 5, 6B, 7F, 8, 9N, 9V, 10A, 11A, 12F, 14, 15B, 17F, 18C, 19A, 19F, 20, 22F, 23F, and 33F. ^c^ New sequence types (STs) identified in this study are shown in bold, and four isolates from blood are show in bold with asterisk. ^d^ MLST, multilocus sequence typing. Gene locus numbers that are different from those of Pneumococcal Molecular Epidemiology Network (PMEN) clones or commn STs (right column) are shown in bold with underline. ^e^ SLV, single locus variant; DLV, double locus variant.

**Table 2:** Prevalence of pneumococcal protein genes in all isolates among individual serotypes.

	No. of Isolates with Pneumococcal Protein Gene																			
Serotype	*pspA^a^*		*pspC*			*pht*							*nan*				Others			
(No. of Isolates)	fam1/fam2/fam3		*pspC*	*pspC.4*		*phtA*	*phtB*	*phtD*	*phtE*	*phtA/B^b^*	*phtA/D^b^*		*nanA*	*nanB*	*nanC*		*pcpA*	*psrp*	*ply*	*pavA*
PCV13 Serotype																				
3 (5)	4/1/0		5	0		1	0	1	5	4	0		5	5	1		1	1	5	5
6A (2)	1/1/0		1	1		1	1	1	2	1	0		2	2	0		2	1	2	2
19F (2)	1/2/0		2	2		0	1	0	2	1	1		2	2	0		2	0	2	2
6B (2)	1/1/0		0	0		0	0	2	2	2	0		2	2	1		2	2	2	2
PPSV23 Serotype (Except PCV13 Type)																				
10A (2)	2/0/0		2	0		2	0	2	2	0	0		2	2	2		2	0	2	2
11A/11D (2)	0/2/0		2	0		2	0	2	2	0	0		2	2	1		2	0	2	2
12F (1)	0/1/0		0	0		0	0	1	1	1	0		1	1	1		1	0	1	1
14 (2)	2/0/0		2	0		0	0	2	2	2	0		2	2	2		2	2	2	2
15B (4)	0/4/0		3	2		0	0	2	4	0	4		4	4	4		4	4	4	4
22F/22A (4)	4/0/0		4	4		0	0	0	4	4	0		4	4	0		4	0	4	4
33F (1)	1/0/0		1	1		1	1	0	1	0	0		1	1	1		1	1	1	1
Non-Vaccine Serotype																				
6C (5)	4/1/0		4	0		4	4	5	5	1	0		5	5	5		5	1	5	5
6E (1)	1/0/0		1	0		1	1	0	1	0	0		1	1	1		1	0	1	1
15A (8)	0/6/0		2	0		7	0	8	8	1	0		8	8	7		7	7	8	8
15C (2)	0/2/0		2	2		0	0	0	2	0	2		2	2	2		2	2	2	2
23A (2)	2/0/0		2	0		2	2	2	2	0	0		2	2	2		2	0	2	2
24F (2)	2/0/0		2	0		2	0	2	2	0	0		2	2	0		2	0	2	2
31 (1)	1/0/0		1	0		1	1	0	1	0	0		1	1	0		1	0	1	1
34 (2)	2/0/0		2	0		1	1	0	2	1	0		2	2	0		2	2	2	2
35B (5)	5/0/0		5	3		1	1	4	5	3	1		5	5	0		5	1	5	5
37 (2)	0/0/2		2	0		2	2	1	2	0	0		2	2	0		2	0	2	2
Total (57)	33/20/2		45	15		28	15	35	57	21	8		57	57	30		52	24	57	57
Positive Rate; %	57.9/35.1/3.5		78.9	26.3		49.1	26.3	61.4	100	36.9	14.0		100	100	52.6		91.2	42.1	100	100

^a^ Family type of PspA: fam1, family1; fam2, family 2; fam3, family 3. ^b^ Fusion type of *pht* genes: *phtA/B*, *phtA* and *phtB*; *phtA/D*, *phtA* and *phtD*.

**Table 3:** Pht profiles of all the *Streptococcus pneumoniae* isolates.

Profile of *pht* Genes	No. of Isolates (%)	Serotypes (No. of Isolates)
*phtA* + *phtB* + *phtE*	5 (8.8)	6E (1), 31 (1), 33F (1), 34 (1), 37 (1)
*phtA* + *phtD* + *phtE*	15 (26.3)	3 (1), 10A (2), 11A/11D (2), 15A (7), 24F (2), 35B (1)
*phtA* + *phtB* + *phtD* + *phtE*	8 (14.0)	6A (1), 6C (4), 23A (2), 37 (1)
*phtA/B*^a^ + *phtE*	11 (19.3)	19F (1), 6A (1), 3 (4), 34 (1), 22F (4)
*phtA/B*^a^ + *phtD* + *phtE*	10 (17.5)	6B (2), 6C (1), 12F (1), 14 (2), 15A (1), 35B (3)
*phtA/D*^b^ + *phtE*	4 (7.0)	15B (2), 15C (2)
*phtA/D*^b^ + *phtB* + *phtE*	2 (3.5)	19F (1), 35B (1)
*phtA/D*^b^ + *phtD* + *phtE*	2 (3.5)	15B (2)
Profile of the *pht* Fusion Type	29 (50.9)	

^a^*phtA/B*, fusion type of *phtA* and *phtB.* ^b^ *phtA/D*, fusion type of *phtA* and *phtD.*

## Supplementary Materials

The following are available online at https://www.mdpi.com/2076-0817/8/4/162/s1, Figure S1: Alignment of PhtA/B (fusion type) and PhtA (a)/PhtB (b) amino acid sequences; Figure S2: Alignment of PhtA/D (fusion type) and PhtA (a)/PhtD (b) amino acid sequences; Figure S3: Amino acid sequence alignment of three B cell epitope regions (I, II, III) of PhtA, PhtB, PhtD, and Pht fusion types (PhtA/B, PhtA/D); Table S1: Resistance gene profiles and antimicrobial susceptibility of the 57 isolates among individual serotypes; Table S2: Percent identity matrix based on nucleotide (upper right) and amino acid (lower left) sequences of *phtA*, *phtB*, *phtD*, *phtE*, and *pht* fusion types; Table S3: GenBank accession numbers assigned for *phtA/B*, *phtA/D*, and *phtB* genes of representative pneumococcal isolates analyzed in the present study.
