# Supplementary material for: Prevalence of Various Vaccine Candidate Proteins in Clinical Isolates of Streptococcus pneumoniae: Characterization of the Novel Pht Fusion Proteins PhtA/B and PhtA/D
Source: Pathogens. 2019 Sep 24;8(4):162. doi: 10.3390/pathogens8040162 (PMC6963846; doi:10.3390/pathogens8040162)
Supplement: Supplementary file 1 [file pathogens-08-00162-s001.zip › Sup.R1/Fig S1. PhtAB.docx]

**Figure S1.** Alignment of PhtA/B (fusion type) and PhtA (a)/PhtB (b) amino acid sequences.

**(a)** Alignment of amino acid sequence of the PhtA/B of representative isolates identified in the present study and PhtA (AF291695), shown in red. The serotype of each isolate is indicated in parenthesis. Asterisk indicates identical amino acid. Putative final amino acid of *phtA*-like region of fusion type PhtA/B is shaded. Positions of B cell epitopes (I, II, and III) described previously [27] are shown in yellow.

PhtA-AF291695(4) MKINKKYLVGSAAALILSVCSYELGLYQARTVKENNRVSYIDGKQATQKTENLTPDEVSK 60

PhtA/B-SP253(12F) MKINKKYLVGSAAALILSVCSYELGLYQARTVKENNRVSYIDGKQATQKTENLTPDEVSK 60

PhtA/B-SP277(3) MKINKKYLVGSAAALILSVCSYELGLYQARTVKENNRVSYIDGKQATQKTENLTPDEVSK 60

PhtA/B-SP224(6B) MKINKKYLVGSAAALILSVCSYELGLYQARTVKENNRVSYIDGKQATQKTENSTPDEVSK 60

PhtA/B-SP272(34) MKINKKYLVGSAAALILSVCSYELGLYQARTVKENNRVSYIDGKQATQKTENLTPDEVSK 60

**************************************************** *******

**Epitope I**

PhtA-AF291695(4) REGINAEQIVIKITDQGYVTSHGDHYHYYNGKVPYDAIISEELLMKDPNYKLKDEDIVNE 120

PhtA/B-SP253(12F) REGINAEQIVIKITDQGYVTSHGDHYHYYNGKVPYDAIISEELLMKDPNYQLKDEDIISE 120

PhtA/B-SP277(3) REGINAEQIVIKITDQGYVTSHGDHYHYYNGKVPYDAIFSEELLMKDPNYKLKDEDIVNE 120

PhtA/B-SP224(6B) REGINAEQIVIKITDQGYVTSHGDHYHYYNGKVPYDAIFSEELLMKDPNYKLKDEDIVNE 120

PhtA/B-SP272(34) REGINAEQIVIKITDQGYVTSHGDHYHYYNGKVPYDAIFSEELLMKDPNYKLKDEDIVNE 120

**************************************:***********:******:.*

**Epitope Ⅱ**

PhtA-AF291695(4) VKGGYVIKVDGKYYVYLKDAAHADNVRTKEEINRQKQEHSQHREGGTPRNDGAVALARSQ 180

PhtA/B-SP253(12F) IKGGYVIKVDGKYYVYLKDAAHADNVRTKEEINRQKQEHSQHREGGTSANDGAVAFARSQ 180

PhtA/B-SP277(3) VKGGYVIKVDGKYYVYLKDAAHADNVRTKEEINRQKQEHSQHREGGTSTNDGAVAFARSQ 180

PhtA/B-SP224(6B) VKGGYVIKVDGKYYVYLKDAAHADNVRTKEEINRQKQEHSQHREGGTSANDGAVAFARSQ 180

PhtA/B-SP272(34) VKGGYVIKVDGKYYVYLKDAAHADNVRTKEEINRQKQEHSQHREGGTPRNDGAVALARSQ 180

:********************************************** ******:****

**Epitope　Ⅲ**

PhtA-AF291695(4) GRYTTDDGYIFNASDIIEDTGDAYIVPHGDHYHYIPKNELSASELAAAEAFLSGRGNLSN 240

PhtA/B-SP253(12F) GRYTTDDGYIFNASDIIEDTGDAYIVPHGDHYHYIPKNELSASELAAAKAFLSGRGNLSN 240

PhtA/B-SP277(3) GRYTTDDGYIFNASDIIEDTGDAYIVPHGDHYHYIPKNELSASELAAAEAFLSGRENLSN 240

PhtA/B-SP224(6B) GRYTTDDGYIFNASDIIEDTGDAYIVPHGDHYHYIPKNELSASELAAAEAFLSGRGNLSN 240

PhtA/B-SP272(34) GRYTTDDGYIFNASDIIEDTGDAYIVPHGDHYHYIPKNELSASELAAAEAFLSGRGNLSN 240

************************************************:****** ****

PhtA-AF291695(4) SRTYRRQNSDNTSRTNWVPSVSNPGTTNTNTSNNSNTNSQASQSNDIDSLLKQLYKLPLS 300

PhtA/B-SP253(12F) SRTYRRQNSDNTSRTNWVPSVSNPGTTNTNTSNNSNTNSQASQSNDIDSLLKQLYKLPLS 300

PhtA/B-SP277(3) LRTYRRQNSDNTPRTNWVPSVSNPGTTNTNTSNNSNTNSQASQSNDIDSLLKQLYKLPLS 300

PhtA/B-SP224(6B) SRTYRRQNSDNTSRTNWVPSVSNPGTTNTNTSNNSNTNSQASQSNDIDSLLKQLYKLPLS 300

PhtA/B-SP272(34) SRTYRRQNSDNTSRTNWVPSVSNPGTTNTNTSNNSNTNSQASQSNDIDSLLKQLYKLPLS 300

*********** ***********************************************

PhtA-AF291695(4) QRHVESDGLVFDPAQITSRTARGVAVPHGDHYHFIPYSQMSELEERIARIIPLRYRSNHW 360

PhtA/B-SP253(12F) QRHVESDGLIFDPAQITSRTARGVAVPHGNHYHFIPYEQMSELEERIARIIPLRYRSNHW 360

PhtA/B-SP277(3) QRHVESDGLIFDPAQITSRTARGVAVPHGNHYHFIPYEQMSELEERIARIIPLRYRSNHW 360

PhtA/B-SP224(6B) QRHVESDGLVFDPAQITSRTARGVAVPHGNHYHFIPYEQMSELEKRIARIIPLRYRSNHW 360

PhtA/B-SP272(34) QRHVESDGLVFDPAQITSRTARGVAVPHGNHYHFIPYEQMSELEKRIARIIPLRYRSNHW 360

*********:*******************:*******.******:***************

**(b)** Alignment of amino acid sequence of the PhtA/B of representative isolates identified in the present study and PhtB (AF318954 strain), shown in blue. The serotype of each isolate is indicated in parenthesis. Asterisk indicates identical amino acid. The initial amino acid of *phtB*-like region of fusion type PhtA/B is shown in blue. Positions of B cell epitopes (I, II, and III) described previously [27] are shown in yellow.

PhtB-AF318954(4) MKINKKYLAGSVAVLALSVCSYELGRYQAGQDKKESNRVAYIDGDQAGQKAENLTPDEVS 60

PhtA/B-SP253(12F) MKINKKYLVGSAAALILSVCSYELGLYQART-VKENNRVSYIDGKQATQKTENLTPDEVS 59

PhtA/B-SP277(3) MKINKKYLVGSAAALILSVCSYELGLYQART-VKENNRVSYIDGKQATQKTENLTPDEVS 59

PhtA/B-SP224(6B) MKINKKYLVGSAAALILSVCSYELGLYQART-VKENNRVSYIDGKQATQKTENSTPDEVS 59

PhtA/B-SP272(34) MKINKKYLVGSAAALILSVCSYELGLYQART-VKENNRVSYIDGKQATQKTENLTPDEVS 59

********.**.*.* ********* *** **.***:****.** **:** ******

**Epitope I**

PhtB-AF318954(4) KREGINAEQIVIKITDQGYVTSHGDHYHYYNGKVPYDAIISEELLMKDPNYQLKDSDIVN 120

PhtA/B-SP253(12F) KREGINAEQIVIKITDQGYVTSHGDHYHYYNGKVPYDAIISEELLMKDPNYQLKDEDIIS 119

PhtA/B-SP277(3) KREGINAEQIVIKITDQGYVTSHGDHYHYYNGKVPYDAIFSEELLMKDPNYKLKDEDIVN 119

PhtA/B-SP224(6B) KREGINAEQIVIKITDQGYVTSHGDHYHYYNGKVPYDAIFSEELLMKDPNYKLKDEDIVN 119

PhtA/B-SP272(34) KREGINAEQIVIKITDQGYVTSHGDHYHYYNGKVPYDAIFSEELLMKDPNYKLKDEDIVN 119

***************************************:***********:***.**:.

**Epitope II**

PhtB-AF318954(4) EIKGGYVIKVNGKYYVYLKDXAHADNIRTKEEIKRQKQERSHNH---NSRADNAVAAARA 177

PhtA/B-SP253(12F) EIKGGYVIKVDGKYYVYLKDAAHADNVRTKEEINRQKQEHSQHREGGTSANDGAVAFARS 179

PhtA/B-SP277(3) EVKGGYVIKVDGKYYVYLKDAAHADNVRTKEEINRQKQEHSQHREGGTSTNDGAVAFARS 179

PhtA/B-SP224(6B) EVKGGYVIKVDGKYYVYLKDAAHADNVRTKEEINRQKQEHSQHREGGTSANDGAVAFARS 179

PhtA/B-SP272(34) EVKGGYVIKVDGKYYVYLKDAAHADNVRTKEEINRQKQEHSQHREGGTPRNDGAVALARS 179

*:********:********* *****:******:*****:*::: . *.*** **:

**Epitope Ⅲ**

PhtB-AF318954(4) QGRYTTDDGYIFNASDIIEDTGDAYIVPHGDHYHYIPKNELSASELAAAEAYWNGKQGSR 237

PhtA/B-SP253(12F) QGRYTTDDGYIFNASDIIEDTGDAYIVPHGDHYHYIPKNELSASELAAAKAFLSGRGNLS 239

PhtA/B-SP277(3) QGRYTTDDGYIFNASDIIEDTGDAYIVPHGDHYHYIPKNELSASELAAAEAFLSGRENLS 239

PhtA/B-SP224(6B) QGRYTTDDGYIFNASDIIEDTGDAYIVPHGDHYHYIPKNELSASELAAAEAFLSGRGNLS 239

PhtA/B-SP272(34) QGRYTTDDGYIFNASDIIEDTGDAYIVPHGDHYHYIPKNELSASELAAAEAFLSGRGNLS 239

*************************************************:*: .*: .

PhtB-AF318954(4) PSSSS-----------SYNANPAQPRLSE-NHNLTVTPTYHQNQGENISSLLRELYAKPL 285

PhtA/B-SP253(12F) NSRTYRRQNSDNTSRTNWVPSVSNPGTTNTNTSNNSNTNSQASQSNDIDSLLKQLYKLPL 299

PhtA/B-SP277(3) NLRTYRRQNSDNTPRTNWVPSVSNPGTTNTNTSNNSNTNSQASQSNDIDSLLKQLYKLPL 299

PhtA/B-SP224(6B) NSRTYRRQNSDNTSRTNWVPSVSNPGTTNTNTSNNSNTNSQASQSNDIDSLLKQLYKLPL 299

PhtA/B-SP272(34) NSRTYRRQNSDNTSRTNWVPSVSNPGTTNTNTSNNSNTNSQASQSNDIDSLLKQLYKLPL 299

: .: . ::* :: * . . . . : .*.::*.***::** **

PhtB-AF318954(4) SERHVESDGLIFDPAQITSRTARGVAVPHGNHYHFIPYEQMSELEKRIARIIPLRYRSNH 345

PhtA/B-SP253(12F) SQRHVESDGLIFDPAQITSRTARGVAVPHGNHYHFIPYEQMSELEERIARIIPLRYRSNH 359

PhtA/B-SP277(3) SQRHVESDGLIFDPAQITSRTARGVAVPHGNHYHFIPYEQMSELEERIARIIPLRYRSNH 359

PhtA/B-SP224(6B) SQRHVESDGLVFDPAQITSRTARGVAVPHGNHYHFIPYEQMSELEKRIARIIPLRYRSNH 359

PhtA/B-SP272(34) SQRHVESDGLVFDPAQITSRTARGVAVPHGNHYHFIPYEQMSELEKRIARIIPLRYRSNH 359

*:********:**********************************:**************

**PhtA-like**

PhtA-AF291695(4) VPDSRPEQPSPQPTPEPSPGPQPAPNLKIDS----NSSLVSQLVRKVGEGYVFEEKGISR 416

PhtA/B-SP253(12F) VPDSRPEQPSP----------QPAPNPQPAPSNPIDEKLVKEAVRKVGDGYVFEENGVSR 410

PhtA/B-SP277(3) VPDSRPEQPSLQPTPEPSPSPQPAPSPQPAPSNPIDEKLVKEAVRKVGDGYVFEENGVSR 420

PhtA/B-SP224(6B) VPDSRPEQPSPQPTPEPSPSPQPAPNPQPAPSNPIDEKLVKEAVRKVGDGYVFEENGVSR 420

PhtA/B-SP272(34) VPDSRPEQPSPQSTPEPSPSLQPAPNPQPAPSNPIDEKLVKEAVRKVGDGYVFEENGVSR 420

********** ****. : :..**.: *****:******:*:**

PhtA-AF291695(4) YVFAKDLPSETVKNLESKLSKQESVSHTLTAKKENVAPRDQEFYDKAYNLLTEAHKALFX 476

PhtA/B-SP253(12F) YIPAKDLSAETAAGIDSKLAKQESLSHKLGAKKTDLPSSDREFYNKAYDLLARIHQDLLD 470

PhtA/B-SP277(3) YIPAKDLSAETAAGIDSKLAKQESLSHKLGAKKTDLPSSDREFYNKAYDLLARIHQDLLD 480

PhtA/B-SP224(6B) YIPAKDLSAETAAGIDSKLAKQESLSHKLGTKKTDLPSSDREFYNKAYDLLARIHQDLLD 480

PhtA/B-SP272(34) YIPAKDLSAETAAGIDSKLAKQESLSHKLGAKKTDLPSSDREFYNKAYDLLARIHQDLLD 480

*: **** :**. .::***:****:**.* :** :: *:***:***:**:. *: *:

PhtA-AF291695(4) NKGRNSDFQALDKLLERLNDESTNKEKLVDDLLAFLAPITHPERLGKPNSQIEYTEDEVR 536

PhtA/B-SP253(12F) NKGRQVDFEALDNLLERLKDVSSDKVKLVEDILAFLAPIRHPERLGKPNSQITYTDDEIQ 530

PhtA/B-SP277(3) NKGRQVDFEALDNLLERLKDVSSDKVKLVDDILAFLAPIRHPERLGKPNAQITYTDDEIQ 540

PhtA/B-SP224(6B) NKGRQVDFEALDNLLERLKDVSSDKVKLVEDILAFLAPIRHPERLGKPNAQITYTDDEIQ 540

PhtA/B-SP272(34) NKGRQVDFEALDNLLERLKDVSSDKVKLVDDILAFLAPIRHPERLGKPNAQITYTDDEIQ 540

****: **:***:*****:* *::* ***:*:******* *********:** **:**::

PhtA-AF291695(4) IAQLADKYTTSDGYIFDEHDIISDEGDAYVTPHMGHSHWIGKDSLSDKEKVAAQAYTKEK 596

PhtA/B-SP253(12F) VAKLAGKYTTEDGYIFDPRDITSDEGDAYVTPHMTHSHWIKKDSLSEAERAAAQAYAKEK 590

PhtA/B-SP277(3) VAKLAGKYTTEDGYIFDPRDITSDEGDAYVTPHMTHSHWIKKDSLSEAERAAAQAYAKEK 600

PhtA/B-SP224(6B) VAKLAGKYTTEDGYIFDPRDITSDEGDAYVTPHMTHSHWIKKDSLSEAERAAAQAYAKEK 600

PhtA/B-SP272(34) VAKLAGKYTTEDGYIFDPRDITSDEGDAYVTPHMTHSHWIKKDSLSEAERAAAQAYAKEK 600

:*:**.****.****** :** ************ ***** *****: *:.*****:***

PhtA-AF291695(4) GILPPSPDADVKANPTGDSAAAIYNRVKGEKRIPLVRLPYMVEHTVEVKNGNLIIPHKDH 656

PhtA/B-SP253(12F) GLTPPSTDHQDSGNTEAKGAEAIYNRVKAAKKVPLDRMPYNLQYTVEVKNGSLIIPHYDH 650

PhtA/B-SP277(3) GLTPPSTGHQDSGNTEAKGAEAIYNRVKAAKKVPLDRMPYNLQYTVEVKNGSLIIPHYDH 660

PhtA/B-SP224(6B) GLTPPSTDHQDSGNTEAKGAEAIYNRVKAAKKVPLDRMPYNLQYTVEVKNGSLIIPHYDH 660

PhtA/B-SP272(34) GLTPPSTDHQDAGNTEAKGAEAIYNRVKAAKKVPLDRMPYNLQYTVEVKNGSLIIPHYDH 660

*: *** . : .* ...* *******. *::** *:** :::*******.***** **

PhtA-AF291695(4) YHNIKFAWFDDHTYKAPNGYTLEDLFATIKYYVEHPDERPHSNDGWGNASEHVLGKKDHS 716

PhtA/B-SP253(12F) YHNIKFEWFDEGLYEAPKGYTLEDLLATVKYYVEHPNERPHSDNGFGNASDHVQRNKNGQ 710

PhtA/B-SP277(3) YHNIKFEWFDEGLYEAPKGYTLEDLLATVKYYVEHPNERPHSDNGFGNASDHVQRNKNGQ 720

PhtA/B-SP224(6B) YHNIKFEWFDEGLYEAPKGYSLEDLLATVKYYVEHPNERPHSDNGFGNASDHVQRNKNGQ 720

PhtA/B-SP272(34) YHNIKFEWFDEGLYEAPKGYTLEDLLATVKYYVEHPNERPHSDNGFGNASDHVQRNKNGQ 720

****** ***: *:**:**:****:**:*******:*****::*:****:** :*: .

PhtA-AF291695(4) EDPNKNF----------------------------------KADEEPVEETPAEPEVPQV 742

PhtA/B-SP253(12F) ADTNQTEKPSEEKPQTEKPEEETPREEKPQSEKPESPKPTEEPEEESPEESPEESEEPQV 770

PhtA/B-SP277(3) ADTNQTEK-----PQTEKPEEETPREEKPQSEKPESPKPTEEP-----EESPEESEEPQV 770

PhtA/B-SP224(6B) ADTNQTEKPNEEKPQTEKPEEETPREEKPQSEKPESPKPTEEP-----EESPEESEEPQV 775

PhtA/B-SP272(34) ADTNQTEKPSEEKPQTEKPEEETPREEKPQSEKPESPKPTEEP-----EESPEESEEPQV 775

* *:. : **:* * * ***

**PhtB-like**

PhtB-AF318954(4) WVPDSRPEEPSPQPTPEPSPSP------QPAPSNPIDGKLVKEAVRKVGDGYVFEENGVS 399

PhtA/B-SP253(12F) WVPDSRPEQPSP----------QPAPNPQPAPSNPIDEKLVKEAVRKVGDGYVFEENGVS 409

PhtA/B-SP277(3) WVPDSRPEQPSLQPTPEPSPSPQPAPSPQPAPSNPIDEKLVKEAVRKVGDGYVFEENGVS 419

PhtA/B-SP224(6B) WVPDSRPEQPSPQPTPEPSPSPQPAPNPQPAPSNPIDEKLVKEAVRKVGDGYVFEENGVS 419

PhtA/B-SP272(34) WVPDSRPEQPSPQSTPEPSPSLQPAPNPQPAPSNPIDEKLVKEAVRKVGDGYVFEENGVS 419

********:** ********* **********************

PhtB-AF318954(4) RYIPAKDLSAETAAGIDSKLAKQESLSHKLGTKKTDLPSSDREFYNKAYDLLARIHQDLL 459

PhtA/B-SP253(12F) RYIPAKDLSAETAAGIDSKLAKQESLSHKLGAKKTDLPSSDREFYNKAYDLLARIHQDLL 469

PhtA/B-SP277(3) RYIPAKDLSAETAAGIDSKLAKQESLSHKLGAKKTDLPSSDREFYNKAYDLLARIHQDLL 479

PhtA/B-SP224(6B) RYIPAKDLSAETAAGIDSKLAKQESLSHKLGTKKTDLPSSDREFYNKAYDLLARIHQDLL 479

PhtA/B-SP272(34) RYIPAKDLSAETAAGIDSKLAKQESLSHKLGAKKTDLPSSDREFYNKAYDLLARIHQDLL 479

*******************************:****************************

PhtB-AF318954(4) DNKGRQVDFEALDNLLERLKDVSSDKVKLVEDILAFLAPIRHPERLGKPNAQITYTDDEI 519

PhtA/B-SP253(12F) DNKGRQVDFEALDNLLERLKDVSSDKVKLVEDILAFLAPIRHPERLGKPNSQITYTDDEI 529

PhtA/B-SP277(3) DNKGRQVDFEALDNLLERLKDVSSDKVKLVDDILAFLAPIRHPERLGKPNAQITYTDDEI 539

PhtA/B-SP224(6B) DNKGRQVDFEALDNLLERLKDVSSDKVKLVEDILAFLAPIRHPERLGKPNAQITYTDDEI 539

PhtA/B-SP272(34) DNKGRQVDFEALDNLLERLKDVSSDKVKLVDDILAFLAPIRHPERLGKPNAQITYTDDEI 539

******************************:*******************:*********

PhtB-AF318954(4) QVAKLAGKYTAEDGYIFDPRDITSDEGDAYVTPHMTHSHWIKKDSLSEAERAAAQAYAXE 579

PhtA/B-SP253(12F) QVAKLAGKYTTEDGYIFDPRDITSDEGDAYVTPHMTHSHWIKKDSLSEAERAAAQAYAKE 589

PhtA/B-SP277(3) QVAKLAGKYTTEDGYIFDPRDITSDEGDAYVTPHMTHSHWIKKDSLSEAERAAAQAYAKE 599

PhtA/B-SP224(6B) QVAKLAGKYTTEDGYIFDPRDITSDEGDAYVTPHMTHSHWIKKDSLSEAERAAAQAYAKE 599

PhtA/B-SP272(34) QVAKLAGKYTTEDGYIFDPRDITSDEGDAYVTPHMTHSHWIKKDSLSEAERAAAQAYAKE 599

**********:*********************************************** *

PhtB-AF318954(4) KGLTPPSTDHQDSGNTEAKGAEAIYNXVKAAKKVPLDRMPYNLQYTVEVKNGSLIIPHYD 639

PhtA/B-SP253(12F) KGLTPPSTDHQDSGNTEAKGAEAIYNRVKAAKKVPLDRMPYNLQYTVEVKNGSLIIPHYD 649

PhtA/B-SP277(3) KGLTPPSTGHQDSGNTEAKGAEAIYNRVKAAKKVPLDRMPYNLQYTVEVKNGSLIIPHYD 659

PhtA/B-SP224(6B) KGLTPPSTDHQDSGNTEAKGAEAIYNRVKAAKKVPLDRMPYNLQYTVEVKNGSLIIPHYD 659

PhtA/B-SP272(34) KGLTPPSTDHQDAGNTEAKGAEAIYNRVKAAKKVPLDRMPYNLQYTVEVKNGSLIIPHYD 659

********.***:************* *********************************

PhtB-AF318954(4) HYHNIKFEWFDEGLYEAPKGYTLEDLLATVKYYVEHPNERPHSDNGFGNASDHVQRNKNG 699

PhtA/B-SP253(12F) HYHNIKFEWFDEGLYEAPKGYTLEDLLATVKYYVEHPNERPHSDNGFGNASDHVQRNKNG 709

PhtA/B-SP277(3) HYHNIKFEWFDEGLYEAPKGYTLEDLLATVKYYVEHPNERPHSDNGFGNASDHVQRNKNG 719

PhtA/B-SP224(6B) HYHNIKFEWFDEGLYEAPKGYSLEDLLATVKYYVEHPNERPHSDNGFGNASDHVQRNKNG 719

PhtA/B-SP272(34) HYHNIKFEWFDEGLYEAPKGYTLEDLLATVKYYVEHPNERPHSDNGFGNASDHVQRNKNG 719

*********************:**************************************

PhtB-AF318954(4) QADTNQTEKPSEEKPQTEKPEEETPREEKPQSEKPESPKPTEEP-----EESPEESEEPQ 754

PhtA/B-SP253(12F) QADTNQTEKPSEEKPQTEKPEEETPREEKPQSEKPESPKPTEEPEEESPEESPEESEEPQ 769

PhtA/B-SP277(3) QADTNQTEK-----PQTEKPEEETPREEKPQSEKPESPKPTEEP-----EESPEESEEPQ 769

PhtA/B-SP224(6B) QADTNQTEKPNEEKPQTEKPEEETPREEKPQSEKPESPKPTEEP-----EESPEESEEPQ 774

PhtA/B-SP272(34) QADTNQTEKPSEEKPQTEKPEEETPREEKPQSEKPESPKPTEEP-----EESPEESEEPQ 774

********* ****************************** ***********

PhtA-AF291695(4) ETEKVEAQLKEAEVLLAKVTDSSLKANATETLAGLRNNLTLQIMDNNSIMAEAEKLLALL 802

PhtA/B-SP253(12F) ETEKVKEKLREAEDLLGKIQDPIIKSNAKETLTGLKNNLLFGTQDNNTIMAEAEKLLALL 830

PhtA/B-SP277(3) ETEKVEEKLREAEDLLGKIQDPIIKSNAKETLTGLKNNLLFGTQDNNTIMAEAEKLLALL 830

PhtA/B-SP224(6B) ETEKVEEKLREAEDLLGKIQDPIIKSNAKETLTGLKNNLLFGTQDNNTIMAEAEKLLALL 835

PhtA/B-SP272(34) ETEKVEEKLREAEDLLGKIQDPIIKSNAKETLTGLKNNLLFGTQDNNTIMAEAEKLLALL 835

*****: :*:*** **.*: * :*:**.***:**:*** : ***:************

PhtA-AF291695(4) KGSNPSSVSKEKIN 816

PhtA/B-SP253(12F) KENK---------- 834

PhtA/B-SP277(3) KESK---------- 834

PhtA/B-SP224(6B) KESK---------- 839

PhtA/B-SP272(34) KESK---------- 839

* .:

PhtB-AF318954(4) VETEKVEEKLREAEDLLGKIQDPIIKSNAKETLTGLKNNLLFGTQDNNTIMAEAEKLLAL 814

PhtA/B-SP253(12F) VETEKVKEKLREAEDLLGKIQDPIIKSNAKETLTGLKNNLLFGTQDNNTIMAEAEKLLAL 829

PhtA/B-SP277(3) VETEKVEEKLREAEDLLGKIQDPIIKSNAKETLTGLKNNLLFGTQDNNTIMAEAEKLLAL 829

PhtA/B-SP224(6B) VETEKVEEKLREAEDLLGKIQDPIIKSNAKETLTGLKNNLLFGTQDNNTIMAEAEKLLAL 834

PhtA/B-SP272(34) VETEKVEEKLREAEDLLGKIQDPIIKSNAKETLTGLKNNLLFGTQDNNTIMAEAEKLLAL 834

******:*****************************************************

PhtB-AF318954(4) LKESK 819

PhtA/B-SP253(12F) LKENK 834

PhtA/B-SP277(3) LKESK 834

PhtA/B-SP224(6B) LKESK 839

PhtA/B-SP272(34) LKESK 839

***.*
