# Supplementary material for: Prevalence of Various Vaccine Candidate Proteins in Clinical Isolates of Streptococcus pneumoniae: Characterization of the Novel Pht Fusion Proteins PhtA/B and PhtA/D
Source: Pathogens. 2019 Sep 24;8(4):162. doi: 10.3390/pathogens8040162 (PMC6963846; doi:10.3390/pathogens8040162)
Supplement: Supplementary file 1 [file pathogens-08-00162-s001.zip › Sup.R1/Fig S2. PhtAD.docx]

**Figure S2.** Alignment of PhtA/D (fusion type) and PhtA (a)/PhtD (b) amino acid sequences.

**(a)** Alignment of amino acid sequence of the PhtA/B of representative isolates identified in the present study and PhtA (AF291695), shown in red. The serotype of each isolate is indicated in parenthesis. Asterisk indicates identical amino acid. Putative final amino acid of *phtA*-like region of fusion type PhtA/D is shaded. Positions of B cell epitopes (I, II, and III) described previously [27] are shown in yellow.

PhtA-AF291695 (4) MKINKKYLVGSAAALILSVCSYELGLYQARTVKENNRVSYIDGKQATQKTENLTPDEVSK 60

PhtA/D-SP237 (15B) MKINKKYLVGSAAALILSVCSYELGLYQARTVKENNRVSYIDGKQATQKTENLTPDEVSK 60

PhtA/D-SP257 (15B) MKINKKYLVGSAAALILSVCSYELGLYQARTVKENNRVSYIDGKQATQKTENLTPDEVSK 60

PhtA/D-SP259 (19F) MKINKKYLVGSAAALILSVCSYELGLYQARTVKENNRVSYIDGKQATQKTENLTPDEVSK 60

PhtA/D-SP284 (35B) MKINKKYLVGSAAALILSVCSYELGLYQARTVKENNRVSYIDGKQATQKTENLTPDEVSK 60

************************************************************

**Epitope I**

PhtA-AF291695 (4) REGINAEQIVIKITDQGYVTSHGDHYHYYNGKVPYDAIISEELLMKDPNYKLKDEDIVNE 120

PhtA/D-SP237 (15B) REGINAEQIVIKITDQGYVTSHGDHYHYYNGKVPYDAIFSEELLMKDPNYKLKDEDIVNE 120

PhtA/D-SP257 (15B) REGINAEQIVIKITDQGYVTSHGDHYHYYNGKVPYDAIFSEELLMKDPNYKLKDEDIVNE 120

PhtA/D-SP259 (19F) REGINAEQIVIKITDQGYVTSHGDHYHYYNGKVPYDAIFSEELLMKDPNYKLKDEDIVNE 120

PhtA/D-SP284 (35B) REGINAEQIVIKITDQGYVTSHGDHYHYYNGKVPYDAIISEELLMKDPNYQLKDEDIISE 120

**************************************:***********:******:.*

**Epitope Ⅱ**

PhtA-AF291695 (4) VKGGYVIKVDGKYYVYLKDAAHADNVRTKEEINRQKQEHSQHREGGTPRNDGAVALARSQ 180

PhtA/D-SP237 (15B) VKGGYVIKVDGKYYVYLKDAAHADNVRTKEEINRQKQEHSQHREGGTSTNDGAVAFARSQ 180

PhtA/D-SP257 (15B) VKGGYVIKVDGKYYVYLKDAAHADNVRTKEEINRQKQEHSQHREGGTSTNDGAVAFARSQ 180

PhtA/D-SP259 (19F) VKGGYVIKVDGKYYVYLKDAAHADNVRTKEEINRQKQEHSQHREGGTSTNDGAVAFARSQ 180

PhtA/D-SP284 (35B) IKGGYVIKVDGKYYVYLKDAAHADNVCTKEEINRQKQEHSQHREGGTSANDGAVAFARSQ 180

:************************* ******************** ******:****

**Epitope Ⅲ**

PhtA-AF291695 (4) GRYTTDDGYIFNASDIIEDTGDAYIVPHGDHYHYIPKNELSASELAAAEAFLSGRGNLSN 240

PhtA/D-SP237 (15B) GRYTTDDGYIFNASDIIEDTGDAYIVPHGDHYHYIPKNELSASELAAAEAFLSGRENLSN 240

PhtA/D-SP257 (15B) GRYTTDDGYIFNASDIIEDTGDAYIVPHGDHYHYIPKNELSASELAAAEAFLSGRENLSN 240

PhtA/D-SP259 (19F) GRYTTDDGYIFNASDIIEDTGDAYIVPHGDHYHYIPKNELSASELAAAEAFLSGRENLSN 240

PhtA/D-SP284 (35B) GRYTTDDGYIFNASDIIEDTGDAYIVPHGDHYHYIPKNELSASELAAAEAFLSGRENLSN 240

******************************************************* ****

PhtA-AF291695 (4) SRTYRRQNSDNTSRTNWVPSVSNPGTTNTNTSNNSNTNSQASQSNDIDSLLKQLYKLPLS 300

PhtA/D-SP237 (15B) LRTYRRQNSDNTPRTNWVPSVSNPGTTNTNTSNNSNTNSQASQSNDIDSLLKQLYKLPLS 300

PhtA/D-SP257 (15B) LRTYRRQNSDNTPRTNWVPSVSNPGTTNTNTSNNSNTNSQASQSNDIDSLLKQLYKLPLS 300

PhtA/D-SP259 (19F) LRTYRRQNSDNTPRTNWVPSVSNPGTTNTNTSNNSNTNSQASQSNDIDSLLKQLYKLPLS 300

PhtA/D-SP284 (35B) LRTYRRQNSDNTPRTNWVPSVSNPGTTNTNTSNNSNTNSQASQSNDIDSLLKQLYKLPLS 300

*********** ***********************************************

PhtA-AF291695 (4) QRHVESDGLVFDPAQITSRTARGVAVPHGDHYHFIPYSQMSELEERIARIIPLRYRSNHW 360

PhtA/D-SP237 (15B) QRHVESDGLIFDPAQITSRTARGVAVPHGNHYHFIPYSQMSELEKRIARIIPLRYRSNHW 360

PhtA/D-SP257 (15B) QRHVESDGLIFDPAQITSRTARGVAVPHGNHYHFIPYSQMSELEKRIARIIPLRYRSNHW 360

PhtA/D-SP259 (19F) QRHVESDGLIFDPAQITSRTARGVAVPHGNHYHFIPYSQMSELEKRIARIIPLRYRSNHW 360

PhtA/D-SP284 (35B) QRHVESDGLIFDPAQITSRTARGVAVPHGNHYHFIPYEQMSELEKRIARIIPLRYRSNHW 360

*********:*******************:*******.******:***************

**(b)** Alignment of amino acid sequence of the PhtA/D of representative isolates identified in the present study and PhtD (AF318955 and KP127692 strains), shown in green. The serotype of each isolate is indicated in parenthesis. Asterisk indicates identical amino acid. The initial amino acid of *phtD*-like region of fusion type PhtA/D is shown in blue. Positions of B cell epitopes (I, II, and III) described previously [27] are shown in yellow.

PhtD-AF318955 (4) MKINKKYLAGSVAVLALSVCSYELGRHQAGQVKKESNRVSYIDGDQAGQKAENLTPDEVS 60

PhtD-KP127692 MKINKKYLAGSVAVLALSVCSYELGRYQAGQDKKESNRVAYIDGDQAGQKAENLTPDEVS 60

PhtA/D-SP237 (15B) MKINKKYLVGSAAALILSVCSYELGLYQARTV-KENNRVSYIDGKQATQKTENLTPDEVS 59

PhtA/D-SP259 (19F) MKINKKYLVGSAAALILSVCSYELGLYQARTV-KENNRVSYIDGKQATQKTENLTPDEVS 59

PhtA/D-SP284 (35B) MKINKKYLVGSAAALILSVCSYELGLYQARTV-KENNRVSYIDGKQATQKTENLTPDEVS 59

********.**.*.* ********* :** **.***:****.** **:*********

**Epitope I**

PhtD-AF318955 (4) KREGINAEQIVIKITDQGYVTSHGDHYHYYNGKVPYDAIISEELLMKDPNYQLKDSDIVN 120

PhtD-KP127692 KREGINAEQIVIKITDQGYVTSHGDHYHYYNGKVPYDAIISEELLMKDPNYQLKDSDIVN 120

PhtA/D-SP237 (15B) KREGINAEQIVIKITDQGYVTSHGDHYHYYNGKVPYDAIFSEELLMKDPNYKLKDEDIVN 119

PhtA/D-SP259 (19F) KREGINAEQIVIKITDQGYVTSHGDHYHYYNGKVPYDAIFSEELLMKDPNYKLKDEDIVN 119

PhtA/D-SP284 (35B) KREGINAEQIVIKITDQGYVTSHGDHYHYYNGKVPYDAIISEELLMKDPNYQLKDEDIIS 119

***************************************:***********:***.**:.

**Epitope Ⅱ**

PhtD-AF318955 (4) EIKGGYVIKVDGKYYVYLKDAAHADNIRTKEEIKRQKQEHSHNHGGG--SNDQAVVAARA 178

PhtD-KP127692 EIKGGYVIKVDGKYYVYLKDAAHADNIRTKEEIKRQKQERSHNHNSR---ADNAVAAARA 177

PhtA/D-SP237 (15B) EVKGGYVIKVDGKYYVYLKDAAHADNVRTKEEINRQKQEHSQHREGGTSTNDGAVAFARS 179

PhtA/D-SP259 (19F) EVKGGYVIKVDGKYYVYLKDAAHADNVRTKEEINRQKQEHSQHREGGTSTNDGAVAFARS 179

PhtA/D-SP284 (35B) EIKGGYVIKVDGKYYVYLKDAAHADNVCTKEEINRQKQEHSQHREGGTSANDGAVAFARS 179

*:************************: *****:*****:*::: . * **. **:

**Epitope Ⅲ**

PhtD-AF318955 (4) QGRYTTDDGYIFNASDIIEDTGDAYIVPHGDHYHYIPKNELSASELAAAEAYWNGKQGSR 238

PhtD-KP127692 QGRYTTDDGYIFNASDIIEDTGDAYIVPHGDHYHYIPKSDLSASELAAAQAYWNGKQGSR 237

PhtA/D-SP237 (15B) QGRYTTDDGYIFNASDIIEDTGDAYIVPHGDHYHYIPKNELSASELAAAEAFLSGRENLS 239

PhtA/D-SP259 (19F) QGRYTTDDGYIFNASDIIEDTGDAYIVPHGDHYHYIPKNELSASELAAAEAFLSGRENLS 239

PhtA/D-SP284 (35B) QGRYTTDDGYIFNASDIIEDTGDAYIVPHGDHYHYIPKNELSASELAAAEAFLSGRENLS 239

**************************************.:*********:*: .*::.

PhtD-AF318955 (4) PSSSSSYNANPAQPRL------SE------NHNLTVTPTYHQNQGENISSLLRELYAKPL 286

PhtD-KP127692 PSSSSSHNANPAQPRL------SE------NHNLTVTPTYHQNQGENISSLLRELYAKPL 285

PhtA/D-SP237 (15B) NLRTYRRQNSDNTPRTNWVPSVSNPGTTNTNTSNNSNTNSQASQSNDIDSLLKQLYKLPL 299

PhtA/D-SP259 (19F) NLRTYRRQNSDNTPRTNWVPSVSNPGTTNTNTSNNSNTNSQASQSNDIDSLLKQLYKLPL 299

PhtA/D-SP284 (35B) NLRTYRRQNSDNTPRTNWVPSVSNPGTTNTNTSNNSNTNSQASQSNDIDSLLKQLYKLPL 299

: : . ** *: * . . . . : .*.::*.***::** **

PhtD-AF318955 (4) SERHVESDGLIFDPAQITSRTARGVAVPHGNHYHFIPYEQMSELEKRIARIIPLRYRSNH 346

PhtD-KP127692 SERHVESDGLIFDPAQITSRTANGVAVPHGDHYHFIPYSQLSPLEEKLARIIPLRYRSNH 345

PhtA/D-SP237 (15B) SQRHVESDGLIFDPAQITSRTARGVAVPHGNHYHFIPYSQMSELEKRIARIIPLRYRSNH 359

PhtA/D-SP259 (19F) SQRHVESDGLIFDPAQITSRTARGVAVPHGNHYHFIPYSQMSELEKRIARIIPLRYRSNH 359

PhtA/D-SP284 (35B) SQRHVESDGLIFDPAQITSRTARGVAVPHGNHYHFIPYEQMSELEKRIARIIPLRYRSNH 359

*:********************.*******:*******.*:* **:::************

**PhtA-like**

PhtA-AF291695 (4) VPDSRPEQPSPQPTPEPSPGPQPAPNLKID----SNSSLVSQLVRKVGEGYVFEEKGISR 416

PhtA/D-SP237 (15B) VPDSRPEQPSPQSTPEPSPSLQPAPNPQPAPSNPIDEKLVKEAVRKVGDGYVFEENGVSR 420

PhtA/D-SP257 (15B) VPDSRPEQPSPQSTPEPSPSLQPAPNPQPAPSNPIDEKLVKEAVRKVGDGYVFEENGVSR 420

PhtA/D-SP259 (19F) VPDSRPEQPSPQSTPEPSPSLQPAPNPQPAPSNPIDEKLVKEAVRKVGDGYVFEENGVSR 420

PhtA/D-SP284 (35B) VPDSRPEQPSPQSTPEPSPSLQPAPNPQPAPSNPIDEKLVKEAVRKVGDGYVFEENGVSR 420

************ ******. ***** : :..**.: *****:******:*:**

PhtA-AF291695 (4) YVFAKDLPSETVKNLESKLSKQESVSHTLTAKKENVAPRDQEFYDKAYNLLTEAHKALFX 476

PhtA/D-SP237 (15B) YIPAKDLSAETAAGIDSKLAKQESLSHKLGAKKTDLPSSDREFYNKAYDLLARIHQDLLD 480

PhtA/D-SP257 (15B) YIPAKDLSAETAAGIDSKLAKQESLSHKLGAKKTDLPSSDREFYNKAYDLLARIHQDLLD 480

PhtA/D-SP259 (19F) YIPAKDLSAETAAGIDSKLAKQESLSHKLGAKKTDLPSSDREFYNKAYDLLARIHQDLLD 480

PhtA/D-SP284 (35B) YIPAKDLSAETAAGIDSKLAKQESLSHKLGAKKTDLPSSDREFYNKAYDLLARIHQDLLD 480

*: **** :**. .::***:****:**.* *** :: *:***:***:**:. *: *:

PhtA-AF291695 (4) NKGRNSDFQALDKLLERLNDESTNKEKLVDDLLAFLAPITHPERLGKPNSQIEYTEDEVR 536

PhtA/D-SP237 (15B) NKGRQVDFEALDNLLERLKDVSSDKVKLVDDILAFLAPIRHPERLGKPNAQITYTDDEIQ 540

PhtA/D-SP257 (15B) NKGRQVDFEALDNLLERLKDVSSDKVKLVDDILAFLAPIRHPERLGKPNAQITYTDDEIQ 540

PhtA/D-SP259 (19F) NKGRQVDFEALDNLLERLKDVSSDKVKLVDDILAFLAPIRHPERLGKPNAQITYTDDEIQ 540

PhtA/D-SP284 (35B) NKGRQVDFEALDNLLERLKDVSSDKVKLVDDILAFLAPIRHPERLGKPNAQITYTDDEIQ 540

****: **:***:*****:* *::* *****:******* *********:** **:**::

PhtA-AF291695 (4) IAQLADKYTTSDGYIFDEHDIISDEGDAYVTPHMGHSHWIGKDSLSDKEKVAAQAYTKEK 596

PhtA/D-SP237 (15B) VAKLAGKYTTEDGYIFDPRDITSDEGDAYVTPHMTHSHWIKKDSLSEAERAAAQAYAKEK 600

PhtA/D-SP257 (15B) VAKLAGKYTTEDGYIFDPRDITSDEGDAYVTPHMTHSHWIKKDSLSEAERAAAQAYAKEK 600

PhtA/D-SP259 (19F) VAKLAGKYTTEDGYIFDPRDITSDEGDAYVTPHMTHSHWIKKDSLSEAERAAAQAYAKEK 600

PhtA/D-SP284 (35B) VAKLAGKYTTEDGYIFDPRDITSDEGDAYVTPHMTHSHWIKKDSLSEAERAAAQAYAKEK 600

:*:**.****.****** :** ************ ***** *****: *:.*****:***

PhtA-AF291695 (4) GILPPSPDADVKANPTGDSAAAIYNRVKGEKRIPLVRLPYMVEHTVEVKNGNLIIPHKDH 656

PhtA/D-SP237 (15B) GLTPPSTDHQDSGNTEAKGAEAIYNRVKAAKKVPLDRMPYNLQYTVEVKNGSLIIPHYDH 660

PhtA/D-SP257 (15B) GLTPPSTDHQDSGNTEAKGAEAIYNRVKAAKKVPLDRMPYNLQYTVEVKNGSLIIPHYDH 660

PhtA/D-SP259 (19F) GLTPPSTDHQDSGNTEAKGAEAIYNRVKAAKKVPLDRMPYNLQYTVEVKNGSLIIPHYDH 660

PhtA/D-SP284 (35B) GLTPPSTDHQDSGNTEAKGAEAIYNRVKAAKKVPLDRMPYNLQYTVEVKNGSLIIPHYDH 660

*: *** * : ..* ...* *******. *::** *:** :::*******.***** **

PhtA-AF291695 (4) YHNIKFAWFDDHTYKAPNGYTLEDLFATIKYYVEHPDERPHSNDGWGNASEHVLGKKDHS 716

PhtA/D-SP237 (15B) YHNIKFEWFDEGLYEAPKGYTLEDLLATVKYYVEHPNERPHSDNGFGNASDHVRKNKVD- 719

PhtA/D-SP257 (15B) YHNIKFEWFDEGLYEAPKGYTLEDLLATVKYYVEHPNERPHSDNGFGNASDHVRKNKVD- 719

PhtA/D-SP259 (19F) YHNIKFEWFDEGLYEAPKGYTLEDLLATVKYYVEHPNERPHSDNGFGNASDHVRKNKVD- 719

PhtA/D-SP284 (35B) YHNIKFEWFDEGLYEAPKGYTLEDLLATVKYYVEHPNERPHSDNGFGNASDHVQRNKNGQ 720

****** ***: *:**:*******:**:*******:*****::*:****:** :*

**PhtD-like**

PhtD-AF318955 (4) WVPDSRPEQPSPQSTPEPSPSPQPAPNPQPAPSNPIDEKLVKEAVRKVGDGYVFEENGVS 406

PhtD-KP127692 WVPDSRPEQPSPQSTPEPSPSPQPAPNPQPAPSNPIDEKLVKEAVRKVGDGYVFEENGVP 405

PhtA/D-SP237 (15B) WVPDSRPEQPSPQSTPEPSPSLQPAPNPQPAPSNPIDEKLVKEAVRKVGDGYVFEENGVS 419

PhtA/D-SP259 (19F) WVPDSRPEQPSPQSTPEPSPSLQPAPNPQPAPSNPIDEKLVKEAVRKVGDGYVFEENGVS 419

PhtA/D-SP284 (35B) WVPDSRPEQPSPQSTPEPSPSLQPAPNPQPAPSNPIDEKLVKEAVRKVGDGYVFEENGVS 419

********************* *************************************

PhtD-AF318955 (4) RYIPAKDLSAETAAGIDSKLAKQESLSHKLGAKKTDLPSSDREFYNKAYDLLARIHQDLL 466

PhtD-KP127692 RYIPAKDLSAETAAGIDSKLAKQESLSHKLGAKKTDLPSSDREFYNKAYDLLARIHQDLL 465

PhtA/D-SP237 (15B) RYIPAKDLSAETAAGIDSKLAKQESLSHKLGAKKTDLPSSDREFYNKAYDLLARIHQDLL 479

PhtA/D-SP259 (19F) RYIPAKDLSAETAAGIDSKLAKQESLSHKLGAKKTDLPSSDREFYNKAYDLLARIHQDLL 479

PhtA/D-SP284 (35B) RYIPAKDLSAETAAGIDSKLAKQESLSHKLGAKKTDLPSSDREFYNKAYDLLARIHQDLL 479

************************************************************

PhtD-AF318955 (4) DNKGRQVDFEALDNLLERLKDVPSDKVKLVDDILAFLAPIRHPERLGKPNAQITYTDDEI 526

PhtD-KP127692 DNKGRQVDFEALDNLLERLKDVSSDKVKLVDDILAFLAPIRHPERLGKPNAQITYTDDEI 525

PhtA/D-SP237 (15B) DNKGRQVDFEALDNLLERLKDVSSDKVKLVDDILAFLAPIRHPERLGKPNAQITYTDDEI 539

PhtA/D-SP259 (19F) DNKGRQVDFEALDNLLERLKDVSSDKVKLVDDILAFLAPIRHPERLGKPNAQITYTDDEI 539

PhtA/D-SP284 (35B) DNKGRQVDFEALDNLLERLKDVSSDKVKLVDDILAFLAPIRHPERLGKPNAQITYTDDEI 539

********************** *************************************

PhtD-AF318955 (4) QVAKLAGKYTTEDGYIFDPRDITSDEGDAYVTPHMTHSHWIKKDSLSEAERAAAQAYAKE 586

PhtD-KP127692 QVAKLAGKYTTEDGYIFDPRDITSDEGDAYVTPHMTHSHWIKKDSLSEAERAAAQAYAKE 585

PhtA/D-SP237 (15B) QVAKLAGKYTTEDGYIFDPRDITSDEGDAYVTPHMTHSHWIKKDSLSEAERAAAQAYAKE 599

PhtA/D-SP259 (19F) QVAKLAGKYTTEDGYIFDPRDITSDEGDAYVTPHMTHSHWIKKDSLSEAERAAAQAYAKE 599

PhtA/D-SP284 (35B) QVAKLAGKYTTEDGYIFDPRDITSDEGDAYVTPHMTHSHWIKKDSLSEAERAAAQAYAKE 599

************************************************************

PhtD-AF318955 (4) KGLTPPSTDHQDSGNTEAKGAEAIYNRVKAAKKVPLDRMPYNLQYTVEVKNGSLIIPHYD 646

PhtD-KP127692 KGLTPPSTDHQDSGNTEAKGAEAIYNRVKAAKKVPLDRMPYNLQYTVEVKNGSLIIPHYD 645

PhtA/D-SP237 (15B) KGLTPPSTDHQDSGNTEAKGAEAIYNRVKAAKKVPLDRMPYNLQYTVEVKNGSLIIPHYD 659

PhtA/D-SP259 (19F) KGLTPPSTDHQDSGNTEAKGAEAIYNRVKAAKKVPLDRMPYNLQYTVEVKNGSLIIPHYD 659

PhtA/D-SP284 (35B) KGLTPPSTDHQDSGNTEAKGAEAIYNRVKAAKKVPLDRMPYNLQYTVEVKNGSLIIPHYD 659

************************************************************

PhtD-AF318955 (4) HYHNIKFEWFDEGLYEAPKGYTLEDLLATVKYYVEHPNERPHSDNGFGNASDHVRKNKVD 706

PhtD-KP127692 HYHNIKFEWFDEGLYEAPKGYSLEDLLATVKYYVEHPNERPHSDNGFGNASDHVQRNKNG 705

PhtA/D-SP237 (15B) HYHNIKFEWFDEGLYEAPKGYTLEDLLATVKYYVEHPNERPHSDNGFGNASDHVRKNKVD 719

PhtA/D-SP259 (19F) HYHNIKFEWFDEGLYEAPKGYTLEDLLATVKYYVEHPNERPHSDNGFGNASDHVRKNKVD 719

PhtA/D-SP284 (35B) HYHNIKFEWFDEGLYEAPKGYTLEDLLATVKYYVEHPNERPHSDNGFGNASDHVQRNKNG 719

*********************:********************************::** .

PhtA-AF291695 (4) EDPNKN-----------------------------------------------FKADEEP 729

PhtA/D-SP237 (15B) ---------QDSKPDEDKGHDEVSEPTHPESDEKENHAGLNPSADNLYKPSTDTEETEEE 770

PhtA/D-SP257 (15B) ---------QDSKPDEDKGHDEVSEPTHPESDEKENHAGLNPSADNLYKPSTDTEETEEE 770

PhtA/D-SP259 (19F) ---------QDSKPDEDKGHDEVSEPTHPESDEKENHAGLNPSADNLYKPSTDTEETEEE 770

PhtA/D-SP284 (35B) ADTNQTEKPQTEKPEEDKEHDEVSEPTHPESDEKENHAGLNPSADNLYKPSTDTEETEEE 780

: **

PhtA-AF291695 (4) VEETPAEPEVPQVETEKVEAQLKEAEVLLAKVTDSSLKANATETLAGLRNNLTLQIMDNN 789

PhtA/D-SP237 (15B) AEDTTDEAEIPQVEHSVINAKIADAEALLEKVTDPSIRQNAMETLTGLKSSLLLGTKDNN 830

PhtA/D-SP257 (15B) AEDTTDEAEIPQVEHSVINAKIADAEALLEKVTDPSIRQNAMETLTGLKSSLLLGTKDNN 830

PhtA/D-SP259 (19F) AEDTTDEAEIPQVEHSVINAKIADAEALLEKVTDPSIRQNAMETLTGLKSSLLLGTKDNN 830

PhtA/D-SP284 (35B) AEDTTDEAEIPQVEHSVINAKIAEAEALLEKVTDSSIRQNAMETLTGLKSSLLLGTKDNN 840

.*:* * *:**** . ::*:: :**.** **** *:: ** ***:**:..* * ***

PhtA-AF291695 (4) SIMAEAEKLLALLKGSNPSSVSKEKIN 816

PhtA/D-SP237 (15B) TISAEVDSLLALLKKSQPVPIQ----- 852

PhtA/D-SP257 (15B) TISAEVDSLLALLKKSQPVPIQ----- 852

PhtA/D-SP259 (19F) TISAEVDSLLALLKKSQPVPIQ----- 852

PhtA/D-SP284 (35B) TISAEVDSLLALLKESQPTPIQ----- 862

:* **.:.****** *:* :.

PhtD-AF318955 (4) ---------------QDSKPDEDKEHDEVSEPTHPESDEKENHAGLNPSADNLYKPSTDT 751

PhtD-KP127692 QADTNQTEKPNEEKPQTEKPEEDKEHDEVSEPTHPESDEKENHAGLNPSADNLYKPSTDT 765

PhtA/D-SP237 (15B) ---------------QDSKPDEDKGHDEVSEPTHPESDEKENHAGLNPSADNLYKPSTDT 764

PhtA/D-SP259 (19F) ---------------QDSKPDEDKGHDEVSEPTHPESDEKENHAGLNPSADNLYKPSTDT 764

PhtA/D-SP284 (35B) QADTNQTEKP-----QTEKPEEDKEHDEVSEPTHPESDEKENHAGLNPSADNLYKPSTDT 774

* .**:*** ***********************************

PhtD-AF318955 (4) EETEEEAEDTTDEAEIPQVENSVINAKIADAEALLEKVTDPSIRQNAMETLTGLKSSLLL 811

PhtD-KP127692 EETEEEAEDTTDEAEIPQVEHSVINAKIAEAEALLEKVTDSSIRQNAVETLTGLKSSLLL 825

PhtA/D-SP237 (15B) EETEEEAEDTTDEAEIPQVEHSVINAKIADAEALLEKVTDPSIRQNAMETLTGLKSSLLL 824

PhtA/D-SP259 (19F) EETEEEAEDTTDEAEIPQVEHSVINAKIADAEALLEKVTDPSIRQNAMETLTGLKSSLLL 824

PhtA/D-SP284 (35B) EETEEEAEDTTDEAEIPQVEHSVINAKIAEAEALLEKVTDSSIRQNAMETLTGLKSSLLL 834

********************:********:********** ******:************

PhtD-AF318955 (4) GTKDNNTISAEVDSLLALLKESQPAPIQ 839

PhtD-KP127692 GTKDNNTISAEVDSLLALLKESQPTPIQ 853

PhtA/D-SP237 (15B) GTKDNNTISAEVDSLLALLKKSQPVPIQ 852

PhtA/D-SP259 (19F) GTKDNNTISAEVDSLLALLKKSQPVPIQ 852

PhtA/D-SP284 (35B) GTKDNNTISAEVDSLLALLKESQPTPIQ 862

********************:***.***
