# Supplementary material for: Prevalence of Various Vaccine Candidate Proteins in Clinical Isolates of Streptococcus pneumoniae: Characterization of the Novel Pht Fusion Proteins PhtA/B and PhtA/D
Source: Pathogens. 2019 Sep 24;8(4):162. doi: 10.3390/pathogens8040162 (PMC6963846; doi:10.3390/pathogens8040162)
Supplement: Supplementary file 1 [file pathogens-08-00162-s001.zip › Sup.R1/Fig S3. Epitope regions.docx]

**Figure S3.** Amino acid sequence alignment of three B cell epitope regions (I, II, III) of PhtA, PhtB, PhtD, and Pht fusion types (PhtA/B, PhtA/D).

Epitope regions I, II, III correspond to pep11, pep17, and pep19, respectively (PhtD amino acid positions 88-107, 172-191, 200-219, respectively) described by Lagousi et al. [27]. Asterisk denotes identical amino acid, while colon and semicolon indicate chemically or structurally similar amino acids.

**Pht-strain(isolate) Epitope I Epitope II Epitope III**

PhtD-AF318955 HYYNGKVPYDAIISEELLMK AVVAARAQGRYTTDDGYIFN GDAYIVPHGDHYHYIPKNEL

PhtD-KP127692 HYYNGKVPYDAIISEELLMK AVAAARAQGRYTTDDGYIFN GDAYIVPHGDHYHYIPKSDL

PhtA-AF291695 HYYNGKVPYDAIISEELLMK AVALARSQGRYTTDDGYIFN GDAYIVPHGDHYHYIPKNEL

PhtB-AF318954 HYYNGKVPYDAIISEELLMK AVAAARAQGRYTTDDGYIFN GDAYIVPHGDHYHYIPKNEL

PhtA/D-SP284 HYYNGKVPYDAIISEELLMK AVAFARSQGRYTTDDGYIFN GDAYIVPHGDHYHYIPKNEL

PhtA/B-SP253 HYYNGKVPYDAIISEELLMK AVAFARSQGRYTTDDGYIFN GDAYIVPHGDHYHYIPKNEL

PhtA/D-SP237 HYYNGKVPYDAIFSEELLMK AVAFARSQGRYTTDDGYIFN GDAYIVPHGDHYHYIPKNEL

PhtA/B-SP272 HYYNGKVPYDAIFSEELLMK AVALARSQGRYTTDDGYIFN GDAYIVPHGDHYHYIPKNEL

************:******* **. **:************* *****************.:*
